# Reading enjoyment amongst non-leisure readers can affect achievement in secondary school

**DOI:** 10.3389/fpsyg.2014.01214

**Published:** 2014-10-27

**Authors:** Suzanne E. Mol, Jelle Jolles

**Affiliations:** Department of Educational Neuroscience and LEARN! Research Institute for Learning and Education, Faculty of Psychology and Education, VU University AmsterdamAmsterdam, Netherlands

**Keywords:** leisure reading, reading enjoyment, mental imagery, sex differences, school achievement, early adolescence

## Abstract

This study aimed to evaluate determinants of differences in leisure reading behavior and school achievement. We specifically examined reading enjoyment, mental imagery, and sex as predictors in a large, age-homogeneous sample of Dutch secondary school students (*N* = 1,071). Results showed that the prevalence of leisure reading was low in both the lower, pre-vocational track (19.5%) and the higher, pre-academic track (32.5%). Boys read even less than girls. Almost all leisure readers enjoyed reading and engaged in mental imagery, i.e., the propensity “to see images” of a written story in the mind’s eye. Overall, boys who did not like to read for leisure had the poorest school performance. Non-leisure readers who reported that they enjoyed reading got higher school grades in the higher educational track. In the lower track, this was the case for girls. Our study findings imply that reading promotion programs should take into account individual differences in sex, achievement level, and reading enjoyment when aiming to decrease the academic achievement gap.

## INTRODUCTION

There is a large achievement gap between the secondary school students who do and those who do not read books during leisure time (OECD, 2010; Mol and Bus, 2011). From sixth grade onward (Jacobs et al., 2002) sex differences become salient as well in this respect, with boys reading less than girls (Coles and Hall, 2002; Logan and Johnston, 2009; Mullan, 2010). However, some of the non-leisure readers perform better than other non-leisure readers, and it is not known which factors are determinants of this variability. In order to develop effective programs to reduce achievement gaps in secondary school, it is imperative to gain insight into the determinants that could explain individual differences within groups that run a higher risk of lower school achievement. Non-leisure readers are such a group. The present paper describes a large-scale survey aimed at evaluating possible determinants and identifying which of these would be suitable for intervention programs.

We conducted our survey study in an age-homogeneous sample of seventh grade students (*N* = 1,071) from the Netherlands. These 12- and 13-year-olds had just made the transition from primary to secondary school. Adjusting to their new academic environment involves dealing with many challenges. The transition is accompanied by a major change in cognitive and social functioning; the school structure, and the nature of the curriculum is different, new subjects have to be learned, the social network changes, and new friends have to be made. Students have to learn to manage, plan, and execute various homework assignments competing for their attention. Consequently, students’ leisure time activities change as well (e.g., Ferguson and Fraser, 1998; Pedersen, 2005). The demands of their new school environment may reduce the time that students have – and/or take – to read for leisure.

It has been found that almost fifty percent of Dutch 15-year-olds report that they never read for pleasure; this was even higher than the international average of 37% (OECD, 2010). It has to be taken into account, however, that while students might not read for leisure, this does not necessarily mean that they *dislike* reading books. Therefore, the current study aimed both to determine the prevalence of leisure reading in seventh grade as well as of the subjective “joy of reading” experienced by leisure versus non-leisure readers. Because of the recent indications that boys and girls may differ in neuropsychological development (e.g., Lenroot and Giedd, 2010) as well as in reading behavior and enjoyment (Coles and Hall, 2002; Jacobs et al., 2002; Chiu and McBride-Chang, 2006; Logan and Johnston, 2009; Mullan, 2010; OECD, 2010), we also investigated sex differences.

A recent meta-analysis showed that leisure readers in College and University graduated high school with higher GPAs than their non-leisure reading peers (Mol and Bus, 2011). A positive spiral of reciprocal causation seems to explain that leisure readers increase skills that are important for their academic success, such as their vocabulary and reading comprehension. Better skilled readers, in turn, are more likely to enjoy what they are reading, to continue reading voluntarily, and to increase their school performance. In general, students who enjoy reading can get absorbed by the narrative world when reading works of fiction (Green and Brock, 2002; Oatley, 2012). Such reading engagement is thought to support the construction of mental situational models that increase story comprehension (Mar, 2004; Oatley, 2011). Successful reading comprehension, in turn, is not only related to reading enjoyment, but also to academic success (Morgan and Fuchs, 2007; Retelsdorf et al., 2011). Therefore, for non-leisure readers in particular, it is important to determine whether their school achievement is or is not related to their general reading enjoyment. If that is the case, this could be a promising insight for interventions. To this end, we hypothesized that non-leisure readers who do enjoy reading get higher school grades than those who do not enjoy reading. We further expected our findings to indicate that girls in this subgroup achieve better than boys.

One ability that seems to affect reading enjoyment and reading comprehension is mental imagery or the propensity of readers to form mental pictures of the written story in “their mind’s eye” (Sadoski and Paivio, 2001). Interestingly, mental imagery is considered to be an essential part of transportation into the narrative world (Green, 2004; Oatley, 2011). It is also thought to enhance reading comprehension, because forming vivid images of a story improves the quality of readers’ mental situational models (Mar, 2004; Yarkoni et al., 2008; Speer et al., 2009; for a review, see De Koning and Van der Schoot, 2013). However, there is individual variability in the vividness with which people are able to picture scenes in “their mind’s eye” (e.g., Cui et al., 2007). This variability seems to be related to experience (Isaac and Marks, 1994; Sadoski and Paivio, 2001; Gennari, 2012). As leisure readers are expected to have more experience with books and with building mental models that support story comprehension, we will examine whether they are more likely to engage in mental imagery than non-leisure readers. Until now, no sex differences have been reported in mental imagery (e.g., Richardson, 1995).

### THE CURRENT STUDY

The reciprocal model of causation suggests that non-leisure readers are at highest risk of poor school achievement (Mol and Bus, 2011). Children who do not read books voluntarily are less likely to enjoy reading and engage less in mental imagery. Hence, this subgroup is most likely to receive relatively low school grades as compared to students who do read for pleasure and enjoy reading. In the Netherlands, this negative spiral could result in an overrepresentation of non-leisure readers in the lower general educational track of secondary school. That is, the Dutch school system is highly stratified from seventh grade onward. The lower, pre-vocational general educational track (i.e., VMBO) takes 4 years and allows students to continue with a vocational education. The higher, pre-academic educational track takes either five (i.e., HAVO) or six (i.e., VWO) years and prepares students for College and/or University. Students are assigned to one of these tracks in their final year of primary school, when they are 10- to 11-years-old on average. Students’ placement is based on their total score on a nationwide test that includes multiple-choice questions that measure students’ aptitude in Dutch language, reading comprehension, math, world orientation (i.e., geography, biology, and history), and study skills (www.government.nl/issues/education). Research has indeed shown that students in the lower track read less frequently, have lower reading comprehension skills, and enjoy reading less than students in the higher track (Gille et al., 2010; The Dutch Inspectorate of Education, 2012). Lower-track students often find reading texts for school boring and challenging, which is probably due to their relatively poor technical reading skills and lack of adequate comprehension strategies (Schram, 2007).

To better understand the consequences of non-leisure reading, it is important that researchers and practitioners acknowledge individual differences in adolescents’ academic opportunities that are strongly related to their leisure reading habits from an early age onward. In non-stratified school systems, study findings may be confounded by achievement differences that can be attributed to students’ leisure reading history, however. The Dutch schooling system, in contrast, offers us an opportunity to look into two groups that are ought to differ in school achievement, but whose within-group differences in schooling situation are relatively small. For example, students’ grades reflect their performance on exams that are adjusted to their respective textbook knowledge, reading skills, and expected level of subject mastery. We expect that reading enjoyment and mental imagery will play a different role in the actual school achievement of students in the higher, pre-academic track as compared to students in the lower, pre-vocational track. That is, it may be particularly important that students enjoy reading in order to succeed in the higher track, because the complexity level of their courses and textbooks may require stronger developed reading abilities than needed in the lower track. Because girls generally are better students than boys (The Dutch Inspectorate of Education, 2012), it is hypothesized that boys who do not enjoy reading run the highest risk of low school performance in the higher track in particular. Importantly, our findings will apply to students attending different educational systems as well. This study could inform researchers and practitioners across the world about the role of reading enjoyment and mental imagery for students who are relatively low versus high achievers.

In sum, this study addressed the following research questions, separately for students in the lower and higher educational track of their first year in secondary school in the Netherlands:

(1) What is the occurrence of reading enjoyment and mental imagery among leisure and non-leisure readers?
(2) To what extent do reading enjoyment, mental imagery, and sex explain differences in the school achievement of non-leisure readers?

## MATERIALS AND METHODS

### PARTICIPANTS

A large, homogeneous group of 1,071 seventh graders from five secondary schools in the Netherlands (548 boys, 523 girls), with a mean age of *M* = 12.54 years (SD = 0.53; *Range*: 11.25–14.75 years) was analyzed. All but 19 students were 12 years old (47.5%) and 13 years old (50.7%). Except for students who repeated or skipped a year in kindergarten (*n* = 39), students who had repeated (*n* = 126) or skipped (*n* = 32) a grade in primary or secondary school (i.e., grade 1 to grade 7) have been excluded from this sample in order to make our group homogeneous with respect to both age and developmental level. Almost all participants had the Dutch nationality (96.7%) and were native speakers (95.8%). Some students (14.7%) had one or two immigrant parents, of whom 59.0% came from western countries. Thirty-four children (3.2%; *n*_12-year-olds_ = 14, *n*_13-year-olds_ = 20) self-reported that they were officially diagnosed with dyslexia.

Participants were in their first year of secondary school. The schools they attended offered the lower and the higher educational tracks in both single-track classrooms (i.e., lower = VMBO; higher = HAVO, and VWO), as well as in combined track classrooms (i.e., lower = VMBO/HAVO; higher = HAVO/VWO). In the latter, combined classroom type, students stream into a single track classroom in grade 8 or 9. Overall, 35% of our participants (*n* = 375; 50.1% boys) were in the lower general educational track, which closely resembles the Dutch national average of 40% (Ministry of Education Culture and Science, 2011). In our study, 65% of the participants (*n* = 696; 51.7% boys) were enrolled in the higher educational track.

### MEASURES

#### Leisure reading

Students were asked to select those activities they engaged in during leisure time. They were presented with a list of ten activities, including reading. The other activities could be categorized into: physical activities (sports, playing outside, acting); social activities (calling friends, online chatting); creative activities (doing arts and crafts, painting); and screen-related activities (playing video games, using the computer and Internet). For the current study, we categorized all students who checked reading as a leisure activity as *leisure readers* (*n* = 299), and those who did not select reading as *non-leisure readers* (*n* = 772).

#### Reading enjoyment

Students were asked to respond to the following statement: “I love to read books (fiction, comic books)” on a 3-point scale. In reading research (e.g., Bennett et al., 2002; Coles and Hall, 2002; Acevedo-Polakovich et al., 2007), a single-item is often used to tap into this construct. In the field of psychology (e.g., Wanous et al., 1997; Gardner et al., 1998; Dollinger and Malmquist, 2009), it has been shown that such an assessment method is also valid and reliable, particularly in large samples. We dichotomized all answers to create a group of *non-enjoyers* (i.e., not at all true), and *reading enjoyers* (i.e., somewhat true, completely true). We decided to combine the latter answer options, because preliminary analyses showed that our findings were comparable for the group who agreed “somewhat” and “completely.”

#### Mental imagery

Students were asked to indicate whether they recognized themselves in the following profile: “While I am reading a story, I use my imagination. I see a film of the story in my mind’s eye, and I see what happens, and what the main character looks like.” This single-item measure has been shown to be a valid estimator of imagery. In a study among 124 lower-track students attending grade 7–9 and 110 of their parents (Mol et al., unpublished data), we found strong correlations with the sum score of ten items of a validated Dutch questionnaire capturing visual, auditory, and social imagery during reading (Tellegen and Frankhuisen, 2002) and our single-item measure: *r*_adolescents_ = 0.57 (*p* < 0.001) and *r*_parents_ = 0.65 (*p* < 0.001). Students who responded “not at all” in this study were assigned to the *non-imagery* group; whereas students who selected “somewhat true” or “completely true” were categorized as *mental imagery users*.

#### School achievement

Final grades (ranging from 1.0 = very bad, to 10.0 = outstanding) for the first semester of the three school subjects “Dutch,” “mathematics,” and “English as a foreign language” were requested for each student. These grades were used to judge school success (Reed et al., 2010), since successful performance in these three subjects is a main goal of Dutch secondary education (Ministry of Education, Culture and Science, 2006). To ensure that the distribution of scores was similar for each school, and to control for possible grading differences across schools, we first standardized children’s average grade within each school. The overall standardized mean school achievement was equal to *M* = 0 and SD = 1 (*range*: -3.50 to 2.87).

Mean grades significantly differed between classroom types (e.g., HAVO = single-track classroom, HAVO/VWO = combined-track classroom). Students attending single-track classrooms received significantly higher mean school grades in both the lower (*n* = 136, *M* = 0.26, SD = 0.89) and higher general educational tracks (*n* = 330, *M* = 0.27, SD = 1.02) than students attending combined classrooms in both tracks (*n*_lower track_ = 224, *M* = -0.41, SD = 0.92; *n*_higher track_ = 256, *M* = -0.13, SD = 0.94). A plausible explanation could be that teachers have to be relatively stricter when assigning good grades to students in combined classrooms, because students’ referral to a single-track classroom with a relatively higher (e.g., VWO) or lower level (e.g., HAVO) at the end of grade 7 or 8 is based on their mean grades. Consequently, we controlled for classroom type in each analysis with school achievement.

#### Demographics

Students reported their sex (boy/girl), birth date, age, educational track, and classroom type in secondary school (VMBO, VMBO/HAVO, HAVO, HAVO/VWO, VWO). They also reported whether they had skipped or repeated a grade in primary or secondary school. They further filled in their own country of birth as well as their parents’, their first language, and whether they were officially diagnosed with dyslexia.

### PROCEDURE

This study was part of the LEERLIJN study, for which ten schools across the Netherlands were recruited. Care was taken to draw the schools from the pool of “mainstream” secondary schools in the Netherlands. In this process, we aimed to ensure that the schools were similar with respect to socio-demographic factors, ethnicity, and educational quality. For the current comparison study, we excluded four schools that only offered the higher educational track, and one school that had classrooms with a heterogeneous combination of all tracks. Consequently, any differences between students from the lower and higher tracks cannot be attributed to differences in school community, because all children attended secondary schools that offered both lower and higher educational tracks.

In their second semester, students individually completed an online survey on a school computer in a classroom setting. A research assistant explained how to access and fill in the survey. Together with the classroom teacher, the assistant made sure that students worked quietly and independently. Response rates were high: Across the five included schools, complete data were collected for 84.2% (*n* = 1,228) of all students who received an informational letter and got parental consent to participate. Teachers’ response rates for providing students’ average school grades was 88.1% (*n* = 944). The online questionnaire format did not allow students to leave any question unanswered, so there were no missing values.

## RESULTS

As is shown in **Table 1**, students from the two general educational tracks differed on all our variables of interest (i.e., leisure reading, reading enjoyment, mental imagery, school achievement). No differences were found on any background variable (i.e., age, sex, ethnicity, first language, dyslexia diagnosis). In the following sections, we will answer our research questions separately for the lower versus higher educational tracks. First, we will look into frequency distributions. Second, within the subgroup of non-leisure readers, we examined the determinants of students’ school achievement by conducting a GLM Univariate ANCOVA, in which we entered reading enjoyment, imagery, and sex as the independent variables, and classroom type as a covariate.

**Table 1:** Distribution of seventh graders over lower versus higher educational tracks in secondary school.

		Educational track			
		Lower (*n* = 375)	Higher (*n* = 696)		*p-*value
Mean age (SD)		12.55 (0.54)	12.53 (0.53)	*t*(1,069) = 0.44	0.664
Sex (%)				χ*^2^*(1) = 0.25	0.619
	Boys	50.1	51.7		
	Girls	49.9	48.3		
Leisure time reading (%)				χ*^2^*(1) = 20.48	<0.001
	Yes	19.5*	32.5*		
	No	80.5	67.5		
Reading enjoyment (%)				χ*^2^*(1) = 20.28	<0.001
	Yes	66.1	78.7		
	No	33.9*	21.3*		
Mental imagery (%)				χ*^2^*(1) = 9.11	0.003
	Yes	88.0	93.4		
	No	12.0*	6.6		
Mean standard school achievement (SD)		-0.15 (0.96)	0.09 (1.01)	*t*(944) = -3.76	<0.001

*-2 < standardized residuals > 2.

### LOWER EDUCATIONAL TRACK

#### Leisure reading differences

In the lower educational track, 375 seventh graders participated. Only 19.5% (*n* = 73) of students indicated that they read during leisure time. Sixty-six percent of all students were reading enjoyers and 88% engaged in mental imagery.

As expected, leisure readers reported significantly more reading enjoyment [χ^2^(1) = 35.84, *p* < 0.001] and mental imagery [χ^2^(1) = 7.36, *p* = 0.007] than non-leisure readers. Specifically, almost all non-enjoyers were non-leisure readers (97.6%; SR = 2.1). There was hardly any student who did not engage in mental imagery and yet was a leisure reader (0.04%; SR = -2.3). In short, leisure readers were very likely to enjoy reading and to engage in mental imagery.

A minority of 12% of all boys and 27% of all girls indicated that they read during their leisure time. Significant sex differences were found in leisure reading [χ^2^(1) = 14.50, *p* < 0.001]: only 30.1% of all leisure readers were boys (SR = -2.4). In addition, more boys did not enjoy reading (SR = 2.0) than girls (SR = -2.1), χ^2^(1) = 12.70, *p* < 0.001 (see **Table 2** for frequency distributions). There were no sex differences in mental imagery [χ^2^(1) = 0.60, *p* = 0.438].

**Table 2:** Sex distribution between general educational tracks when comparing leisure time readers with non-leisure readers on reading enjoyment and mental imagery.

			Reading enjoyment				Mental imagery			
Educational track	Sex	Leisure reader	No		Yes		No		Yes	
			*n*	%	*n*	%	*n*	%	*n*	%
Lower	Boys	Yes	2	9.1	20	90.9	1	4.5	21	95.5
		No	78	47.0	88	53.0	24	14.3	142	85.5
	Girls	Yes	1	2.0	50	98.0	1	2.0	50	98.0
		No	46	33.8	90	66.2	19	14.0	117	86.0
Higher	Boys	Yes	2	2.9	68	97.1	3	4.3	67	95.7
		No	108	37.2	182	62.8	29	10.0	261	90.0
	Girls	Yes	0	.0	156	100.0	2	1.3	154	98.7
		No	38	21.1	142	78.9	12	6.7	168	93.3

#### School achievement differences among non-leisure readers

In the next analysis, we only included non-leisure readers (*n* = 302). As is shown in **Table 3**, almost all non-leisure readers who enjoyed reading engaged in mental imagery (SR = 3.4), whereas non-leisure readers who did not enjoy reading were significantly more likely not to engage in mental imagery (SR = -2.8), χ^2^(1) = 23.06, *p* < 0.001.

**Table 3:** Differences in reading enjoyment and mental imagery for non-leisure readers in lower and higher general educational tracks.

			Mental imagery				
Educational track	Sex	Reading enjoyment	No		Yes		
			*n*	*%*	*n*		*%*
Lower	Boys	No	18	23.1	60		76.9
		Yes	6	6.8	82		93.2
	Girls	No	14	30.4	32		69.6
		Yes	5	5.6	85		94.4
	Total	No	32	25.8	92		74.2
		Yes	11	6.2	167		93.8
Higher	Boys	No	15	13.9	93		86.1
		Yes	14	7.7	168		92.3
	Girls	No	7	18.4	31		81.6
		Yes	5	3.5	137		96.5
	Total	No	22	15.1	124		84.9
		Yes	19	5.9	305		94.1

**FIGURE 1:**
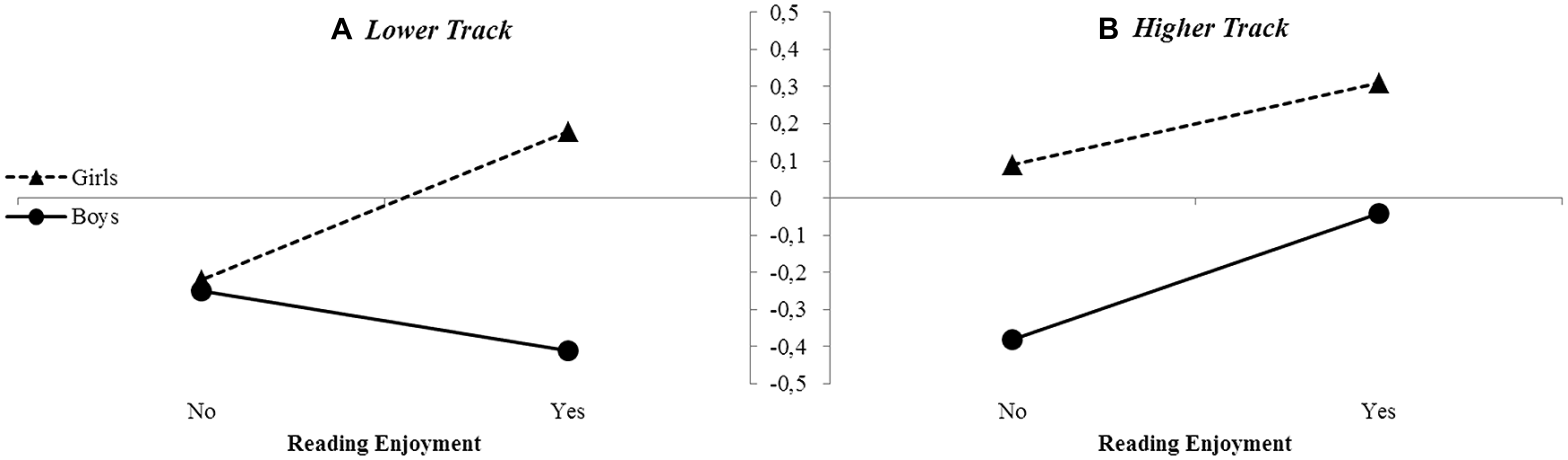
**School achievement differences explained by sex and reading enjoyment in non-leisure readers, for the lower **(A)** and higher **(B)** general educational tracks**.

We conducted a GLM Univariate ANCOVA with school achievement as the dependent variable, and tested main effects of reading enjoyment, mental imagery, and sex as well as the interactions between sex and reading enjoyment, and sex and imagery. Mean grades were available for 290 students. The overall model with these two interaction terms explained 16.8% of the variance [*F*(6,283) = 10.74, *p* < 0.001, η^2^ = 0.19). The main effects for sex [*F*(1,283) = 3.97, *p* = 0.053, η^2^= 0.01], reading enjoyment [*F*(1,283) = 1.41, *p* = 0.235, η^2^= 0.00], and mental imagery [*F*(1,283) = 0.53, *p* = 0.467, η^2^= 0.00) were not significant. The covariate classroom type was [*F*(1,283) = 42.60, *p* < 0.001, η^2^= 0.13].The interaction between sex and reading enjoyment was significant as well [*F*(1,283) = 6.41, *p* = 0.012, η^2^= 0.02]. As is shown in **Figure 1A**, girls who were reading enjoyers scored 0.40 average grade points higher than girls who were non-enjoyers; whereas boys’ reading enjoyment did not seem to explain their school achievements. In short, reading enjoyment appears to play a role in the school achievement of girls in particular.

### HIGHER EDUCATIONAL TRACK

#### Leisure reading differences

Self-report data were collected for 696 students in the higher educational track. A minority of 32.5% (*n* = 226) of seventh graders indicated that they read during their leisure time. About 80% enjoyed reading and 93.4% engaged in mental imagery (see **Table 1**).

Leisure readers were significantly more likely to enjoy reading [χ*^2^*(1) = 83.02, *p* < 0.001] and to engage in mental imagery [χ^2^(1) = 10.48, *p* = 0.001] than non-leisure readers. Specifically, the majority of non-enjoyers were categorized as non-leisure readers (98.6%; SR = 4.6). In addition, the subset of students who did not engage in mental imagery (*n* = 46) were hardly categorized as leisure readers (0.02%; SR = -2.6). Put differently, most leisure readers enjoyed reading and engaged in mental imagery.

Nineteen percent of all boys and 46% of all girls were leisure readers. Of all leisure readers, 31.0% were boys. Significant sex differences were found in leisure reading [χ^2^(1) = 57.71, *p* < 0.001) and reading enjoyment [χ^2^(1) = 38.45, *p* < 0.001]: Boys were underrepresented as leisure readers (SR = -4.3) and reading enjoyers (SR = -2.0), but more likely to be categorized as non-leisure readers (SR = 3.0) and non-enjoyers (SR = 3.8). The opposite pattern was found for girls (SR_leisure readers_ = 4.5, SR_enjoyers_ = 2.1; SR_non-leisure readers_ = -3.1, SR_non-enjoyers_ = -4.0, respectively). This implies that more girls than boys were leisure readers and reading enjoyers (see **Table 2**). Finally, the standardized residuals did not exceed the critical range for mental imagery [i.e., were -1.7, 1.7; χ^2^(1) = 6.28, *p* = 0.012], showing no sex differences.

#### School achievement differences among non-leisure readers

Most non-leisure readers (*n* = 470) enjoyed reading (68.9%) and engaged in mental imagery (91.3%). Those who did not engage in mental imagery were most likely to be categorized as non-enjoyers of reading (SR = 2.6), χ^2^(1) = 10.71, *p* = 0.001 (see **Table 3**).

Mean school grades were available for 400 non-leisure readers in the higher track. GLM Univariate ANCOVA analyses showed that sex, reading enjoyment, mental imagery, and classroom type explained 7.8% of the variance of school achievement [*F*(6,393) = 6.44, *p* < 0.001, η^2^= 0.09]. The interaction between sex and reading enjoyment was not significant [*F*(1,393) = 0.01, *p* = 0.938, η^2^ = 0.00]. Further, the interaction between sex and mental imagery only approached significance [*F*(1,393) = 3.48, *p* = 0.059, η^2^ = 0.01] and will not be explored further due to the relatively small percentage (*n* = 31) of students who did not engage in mental imagery.

Main effects were found for sex [*F*(1,393) = 13.40, *p* < 0.001, η^2^= 0.03] and reading enjoyment [*F*(1,393) = 4.92, *p* = 0.027, η^2^ = 0.01] and the covariate classroom type [*F*(1,393) = 8.75, *p* = 0.003, η^2^= 0.02], but not for mental imagery [*F*(1,393) = 0.07, *p* = 0.791, η^2^= 0.00]. Girls who were reading enjoyers scored 0.22 standardized, average grade points higher than girls who were non-enjoyers; and boys who were reading enjoyers scored 0.34 grade points higher than boys who were non-enjoyers (see **Figure 1B**). Overall, analyses showed that reading enjoyment played a positive role in the school achievement scores of both boys and girls.

## DISCUSSION

This study offered the unique opportunity to examine which determinants of leisure reading are related to school achievement in a large number of students who were similar in educational stage. This similarity reduced variability related to age and schooling. Two research questions were addressed for both the 375 students in the lower track and the 696 students in the higher track of the participating Dutch schools. First, we found that a minority of only 19.5% of students in the lower and 32.5% in the higher educational track indicated that they read during their leisure time. Almost all these leisure readers enjoyed reading and formed images of the story they were reading in “the mind’s eye” (i.e., they engaged in mental imagery). As expected, more girls than boys read outside school and enjoyed reading, whereas no sex differences were found for mental imagery. Second, the majority of non-leisure readers reported that they enjoyed reading. Except for boys in the lower track, this subgroup of reading enjoyers performed better academically than those who did not enjoy reading.

Secondary school students are not a homogeneous group in terms of school achievement and leisure reading behavior. The advantage of a highly stratified educational system like the one in the Netherlands is that we can remove some heterogeneity by separately examining relatively low and high achievers attending the lower and higher educational tracks, respectively. In this study, we found that a greater percentage of students in the lower track were non-leisure readers, did not enjoy reading, and did not engage in mental imagery compared to students in the higher tracks. Lower-track students also got lower school grades on average. These findings highlight the importance of looking into subgroups of secondary school students when aiming to understand the relation between leisure reading and school achievement; a relation that is affected by adolescents’ reading history as well as the quality of their home literacy environment from an early age onward (Conlon et al., 2006). Such an approach can inform interventions that focus on students who run the highest risk of poor school achievement within relatively lower- and higher-achieving groups.

The first aim of this study was to examine the occurrence of leisure reading, reading enjoyment, and mental imagery of 12- and 13-year-old Dutch secondary school students. We found that the majority of students in both educational tracks did not list reading among their leisure activities. More specifically, eight out of ten lower-track students and two out of three higher-track students did not engage in leisure reading. We investigated reading as an integral part of students’ leisure time activities instead of using a question format that is likely to elicit more socially desirable answers, such as estimating reading frequencies (Mol and Bus, 2011). Our approach seemed to reveal an even more skewed distribution of adolescent readers than was found in the recent PISA-investigation, in which 49% of Dutch fifteen-year-old students indicated that they never read for pleasure (Gille et al., 2010; OECD, 2010). Interestingly, more than half of the non-leisure readers in our sample were willing to admit that they enjoyed reading books and comics in general.

Non-leisure readers who did not enjoy reading hardly used mental imagery strategies during reading; whereas those who did read for leisure were almost all imagery users. In both educational tracks, about 94% of the non-leisure readers who indicated that they enjoy reading in general also engaged in mental imagery. These findings seem to be in line with previous research linking reading habits, mental imagery, and reading enjoyment, as part of the experience of transportation into the narrative world (e.g., Green, 2004; Busselle and Bilandzic, 2008; Oatley, 2011; Weibel et al., 2011). In addition, recent theories of embodied cognition (Barsalou, 2008; Glenberg, 2011) propose that reading about a situation in a novel or a story drives the brain into perceptual, active, and emotional states that simulate the mental states that arise during the perception of, and the acting in, an exact same real-life situation. For example, neuroscientific studies show that direct speech statements in a story activate the auditory cortex (Yao et al., 2011), and that reading about happy events elicits greater activity in the muscles that control smiling (Havas et al., 2010). Reading a sentence that implies fictive motion (e.g., The road runs along the coast) produces corresponding simulations of motion through space in the brain (Matlock, 2004). Such simulation is thought to make reading an enjoyable experience (Oatley, 1999; Mar and Oatley, 2008). Future studies should examine whether mental imagery or its vividness is a prerequisite for, or a consequence of, reading enjoyment, or whether these two aspects are reciprocally related. It may further be interesting to examine the role of mental imagery across various school topics (e.g., math, science), as well as across media. Recent studies with adults show that high levels of vividness may enhance the enjoyment of reading books but not of watching movies (e.g., Green et al., 2008; Weibel et al., 2011), but it is not yet known whether this applies equally to early adolescents.

It could seem discouraging that students who just made the transition to secondary school are not very likely to read outside school. However, it is promising that plenty of non-leisure readers in our study did admit that they enjoy reading in general. Fifty-nine percent of these students in the lower educational track and 69% in the higher track claimed to enjoy reading. In this subgroup of non-leisure readers, therefore, it may not be fruitful to stimulate leisure reading through aiming to affect their “subjective joy of reading.” This seems to be in line with models that link actual behavior with motivation (for reviews see, for example, Conner and Armitage, 1998; Eccles and Wigfield, 2002), also within the domain of reading (e.g., Guthrie et al., 2012; Schiefele et al., 2012). One explanation for our current finding could be that students’ transition to secondary school has changed the way they spend their leisure time so that they stopped reading voluntarily. In future research, it would be interesting to explore whether children think that they read less or more than they did in primary school and if so, whether they could explain why this is the case. Students’ school transition does not necessarily have to change their enjoyment of reading books yet, however. According to the negative spiral of reading behavior, it can be speculated that this subgroup of non-leisure readers will lose their current reading enjoyment over the course of their secondary school career; their lack of reading practice may result in less reading enjoyment and decreasing school grades. As long as children admit to enjoying reading in general, it may be particularly important, therefore, that they learn how to effectively structure their time outside school. Early adolescents may particularly need assistance with creating enjoyable reading opportunities, as they are known to be undergoing profound changes in neuropsychological and brain development (e.g., Shaw et al., 2006; Giedd, 2008; Crone and Dahl, 2012). Research shows that parents are still important role models, who can actively guide their early adolescents in their activities or who can help them select materials that match their interests and reading level (Love and Hamston, 2004; Klauda, 2009).

The second aim of this study was to look into the role of sex, reading enjoyment, and mental imagery in the school achievement of non-leisure readers. In the higher track, boys and girls who did not enjoy reading got significantly lower grades than their same-sex peers who did enjoy reading. This also was the case for girls in the lower track. For these boys and girls, it may be interesting to examine whether their school achievement will improve after enhancing their reading enjoyment. Experimental studies in classroom settings are yet limited, but there is some evidence in lower grade levels that programs including a motivational aspect improve students’ reading engagement (e.g., Souvignier and Mokhlesgerami, 2006; Guthrie et al., 2007; DeNaeghel et al., 2013). For example, instructional practices that focus on the relevance of a text, students’ own choice, reading success, collaborative structures, and thematic units seem to increase important motivational processes as intrinsic motivation, perceived autonomy, self-efficacy, social motivation, and mastery goals, respectively (Guthrie et al., 2007). Another approach would be to teach mental imagery strategies, which may affect students’ reading comprehension (Hibbing and Rankin-Erickson, 2003; Algozzine and Douville, 2004; for a review, see De Koning and Van der Schoot, 2013). In line with previous research (Green and Brock, 2002; Oatley, 2012), our study suggests that such an intervention may also benefit students’ reading enjoyment. The positive spiral of reading behavior implies that improving the reading enjoyment in this subgroup of non-enjoyers could enhance their leisure reading behavior and, hence, their school achievement.

Our study highlights that boys are in particular need of successful reading interventions. Almost nine out of ten boys in the lower educational track and eight out of 10 boys in the higher educational track were categorized as non-leisure readers. Interestingly, the sex distribution of reading enjoyers in our sample was rather equal among non-leisure readers: about half of the non-leisure readers who enjoyed reading were boys, in both the lower (49%) and higher (56%) educational tracks. The book market already seems to have developed ways to address the fact that boys may need more stimulation than girls to engage in reading activities. For example, numerous websites can be found online that list books that are considered of specific interest for boys, by classifying them on a range of topics (e.g., animals, war, robots, outer space, sports) or genres (e.g., science fiction, fantasy, young adult) that boys are thought to be attracted to. Some studies suggest that parents and teachers should be encouraged to develop a broader perspective on the range of (online) texts that they would count as “appropriate reading materials” in order for boys to start reading (Telford, 1999; Love and Hamston, 2004). It should be examined, however, whether reading texts in newspapers, magazines, blogs, and/or on informative websites could equally impact students’ reading enjoyment, mental imagery, and reading achievement as reading works of fiction (e.g., Oatley, 1999; Mar et al., 2006). Students seem to particularly be engaged in their reading activity when they are absorbed in the world of the book. The positive spiral of reading suggests that such absorption is needed to continue reading voluntarily.

Our study further provides insight in the group that run the highest risk of poor school performance. Unexpectedly, the reading enjoyment of male non-leisure readers in the lower track was not related to their school achievement. Boys who claimed to enjoy reading got comparable grades as boys and girls who did not enjoy reading. It could be that their reading abilities are so relatively poor that their general reading enjoyment cannot function as a protective factor for low school grades. The current reading abilities of these low achievers may not be sufficient for understanding the age-appropriate reading materials that are supposed to be fun for them to read voluntarily. Books that match their reading level are often written for younger children (Schram, 2007). Either reading promotion initiative is likely to result in a further avoidance of leisure reading, which will further increase their risk of dropping out of secondary or tertiary education. It may be necessary to first train these students’ (technical) reading skills. Put differently, we expect that students need a minimum level of reading abilities before their school achievement will be affected by their general reading enjoyment. Future studies should reveal whether this indeed is the case.

We found no direct relation between mental imagery and school achievement. This finding should be interpreted with caution, however, due to the ceiling effect that seemed to affect our mental imagery measure. To replicate and extend our findings, a questionnaire should be used that captures more variability and modalities in mental imagery behavior (see, for example, Blajenkova et al., 2006). Furthermore, our current indicator of reading enjoyment may have resulted in a dichotomous split that neglected the nuance of the group of students who do not really hate books but who also do not enjoy them as much as their peers who really love reading. Future studies that embed reading enjoyment into the broader concepts of reading motivation, attitude, and interest may reveal a more comprehensive picture as well (Conradi et al., 2014). In addition, we looked at students’ average school grades instead of their reading abilities. Stronger relations could be expected if we had measured students’ actual reading performance. Because reading abilities are an important indicator of general academic success (e.g., Alexander et al., 2007), it is plausible that students with higher grades in our sample also are the better readers. Due to the correlational nature of our study, however, we cannot be sure whether those who did not enjoy reading are the low-achieving students, or whether the low achievers particularly are low reading enjoyers. Learning more about the development of low school achievement, low reading enjoyment and their interaction from an early age onward may particularly be useful when identifying the best approach for reaching students with reading stimulation programs. Interestingly, a peer culture of reading encouragement in schools seems to enhance the reading achievement of both boys and girls, regardless of their reading proficiency (Chiu and McBride-Chang, 2006).

In summary, our study indicates that we have to keep in mind that adolescents’ behaviors may not reflect their motivation and emotional responses toward reading. That is, we identified a group of seventh grade students who enjoy reading even when reading is not among their preferred leisure activities. Accordingly, the present data imply that reading enjoyment could make a difference in students’ school careers. Indeed, we showed that girls who were non-leisure readers and enjoyed reading in general got higher average school grades in both educational tracks. Boys who enjoyed reading in the higher educational track also excelled compared to boys who did not enjoy reading. For their classmates who do not enjoy reading, it could be expected that their school achievement benefits from higher reading enjoyment levels. Boys in the lower track, however, are not likely to profit from such an intervention. This study, therefore, underscores the importance of acknowledging individual differences in sex and reading enjoyment when aiming to improve the academic careers of higher versus lower achieving students.

## Conflict of Interest Statement

The authors declare that the research was conducted in the absence of any commercial or financial relationships that could be construed as a potential conflict of interest.
